# Trans-Differentiation of Neural Stem Cells: A Therapeutic Mechanism Against the Radiation Induced Brain Damage

**DOI:** 10.1371/journal.pone.0025936

**Published:** 2012-02-10

**Authors:** Kyeung Min Joo, Juyoun Jin, Bong Gu Kang, Se Jeong Lee, Kang Ho Kim, Heekyoung Yang, Young-Ae Lee, Yu Jin Cho, Yong-Seok Im, Dong-Sup Lee, Do-Hoon Lim, Dong Hyun Kim, Hong-Duck Um, Sang-Hun Lee, Jung-II Lee, Do-Hyun Nam

**Affiliations:** 1 Department of Anatomy, Seoul National University College of Medicine, Chongno-Gu, Seoul, Korea; 2 Cancer Stem Cell Research Center, Samsung Medical Center and Samsung Biomedical Research Institute, Sungkyunkwan University School of Medicine, Gangnam-gu, Seoul, Korea; 3 Department of Neurosurgery, Samsung Medical Center and Samsung Biomedical Research Institute, Sungkyunkwan University School of Medicine, Gangnam-gu, Seoul, Korea; 4 Department of Radiation Oncology, Samsung Medical Center and Samsung Biomedical Research Institute, Sungkyunkwan University School of Medicine, Gangnam-gu, Seoul, Korea; 5 Center for Molecular and Cellular Imaging, Samsung Medical Center and Samsung Biomedical Research Institute, Sungkyunkwan University School of Medicine, Gangnam-gu, Seoul, Korea; 6 Laboratory of Radiation Tumor Physiology, Korea Institute of Radiological and Medical Sciences, Nowon-gu, Seoul, Korea; 7 Department of Biochemistry and Molecular Biology, College of Medicine, Hanyang University, Seongdong-gu, Seoul, Korea; Stanford University, United States of America

## Abstract

Radiation therapy is an indispensable therapeutic modality for various brain diseases. Though endogenous neural stem cells (NSCs) would provide regenerative potential, many patients nevertheless suffer from radiation-induced brain damage. Accordingly, we tested beneficial effects of exogenous NSC supplementation using *in vivo* mouse models that received whole brain irradiation. Systemic supplementation of primarily cultured mouse fetal NSCs inhibited radiation-induced brain atrophy and thereby preserved brain functions such as short-term memory. Transplanted NSCs migrated to the irradiated brain and differentiated into neurons, astrocytes, or oligodendrocytes. In addition, neurotrophic factors such as NGF were significantly increased in the brain by NSCs, indicating that both paracrine and replacement effects could be the therapeutic mechanisms of NSCs. Interestingly, NSCs also differentiated into brain endothelial cells, which was accompanied by the restoration the cerebral blood flow that was reduced from the irradiation. Inhibition of the VEGF signaling reduced the migration and trans-differentiation of NSCs. Therefore, trans-differentiation of NSCs into brain endothelial cells by the VEGF signaling and the consequential restoration of the cerebral blood flow would also be one of the therapeutic mechanisms of NSCs. In summary, our data demonstrate that exogenous NSC supplementation could prevent radiation-induced functional loss of the brain. Therefore, successful combination of brain radiation therapy and NSC supplementation would provide a highly promising therapeutic option for patients with various brain diseases.

## Introduction

For many central nervous system diseases including brain tumors [1], [2] and arteriovenous malformations [3], treatment options are very limited. Surgical intervention is not viable due to the limited accessibility of the disease location, as well as the high risk of disturbing vital normal brain functions. The use of systemic chemotherapeutics is also ineffective because of the largely impermeable blood-brain barrier (BBB). For example, recent advances in chemotherapies have led to a relatively better control of primary tumors, but they still fail to treat metastasis to the brain since they do not cross the BBB [1], [2], [4], [5], [6]. Since new systemic treatment options become available that increase the longevity of patients with advanced disease [1], the current annual incidence rate of 170,000 new brain metastases in the United States [7] is likely to increase rapidly. High-dose brain radiation therapy is the primary choice for treating both primary and metastatic brain tumors [1], [2], [4], [5]. Taken together, radiation therapy remains as the only remaining indispensable treatment modality.

In contrast to chemotherapy, radiation therapy has the advantage of being local and organ-specific. Recent technical advances such as three-dimensional conformal radiotherapy (3DCRT), intensity-modulated radiation therapy (IMRT), and gamma knife radiosurgery (GKS) allow even further localized and concentrated treatment. Despite these advances, however, exposure of normal brain tissues to detrimental effects of radiation is still unavoidable [4], [5]. Furthermore, certain cases, such as diffuse primary brain tumors or brain metastases with multiple lesions, require the use of whole-brain radiation therapy (WBRT) that would inevitably lead to the exposure of normal tissues to high-dose radiation. Normal brain tissues are sensitive to radiation [4], [8]–[10], and the consequential brain damage is a severe drawback for the use of radiation therapy – one study reported eleven percent of patients receiving high-dose WBRT suffered radiation-induced dementia [11], while others reported significant visual defects, dysarthria, and gait disturbances [4], [8], [10]. Therefore, treatment modalities that can lessen or even prevent radiation-induced brain damage are imperative to make radiation therapy more appealing for clinical use and to improve the quality of life of patients.

It is reported that endogenous neural stem cells (NSCs) provide regenerative potential to irradiated brain tissues [12]–[14]. An attractive hypothesis states that NSCs restore the lost functions of damaged tissues. However, the scarcity of the endogenous NSCs and the extensive degree of damage due to radiation make endogenous restoration impractical. It is therefore necessary to transplant substantial amount of exogenous NSCs to make the cell-based therapy a viable treatment option. Since NSCs are one of the most amenable cell sources for neural transplantation [15], [16], are endowed with extensive functional stability and plasticity [12]–[14], and can be expanded long-term *in vitro* [17], we hypothesized that exogenous supplementation of NSCs could rescue irradiated brains from functional loss. First, we focused on the WBRT since radiation exposure of normal brain tissue is higher than those of focused radiation therapies. It is also clinically relevant since it is the common procedure for treating the increasing number of brain metastasis patients. Here, we demonstrated the beneficial effects of NSC supplementation and elucidated its potential therapeutic mechanisms. Then, we employed animal models that had focused radiation therapy, i.e. GKS, and confirmed the therapeutic mechanisms of NSC supplementation.

## Results

### 1. Primary culture of mouse fetal NSCs expressing green fluorescent protein (GFP^+^ NSCs)

GFP^+^ NSCs were primarily cultured from brains of 13.5 day old GFP transgenic C57BL/6 mouse embryos [18]. In the NSC culture condition without serum (NeuroBasal Media supplemented with N2, B27 and EGF), they grew as neurospheres that expressed NSC markers such as Nestin, Musashi, Sox2, and CD133 (Fig. S1A, B). When NSCs were maintained in 10% FBS/DMEM for two weeks, they showed differentiated neural cell morphologies, lost the expression of the NSC markers, and expressed specific markers of differentiated neural cells such as Tuj1 (neuron), GFAP (astrocyte), or Olig2 (oligodendrocyte) confirming their differential potential (Fig. S1C, D, E).

### 2. Protective effects of NSCs against the radiation-induced brain damage

**Figure 1:**
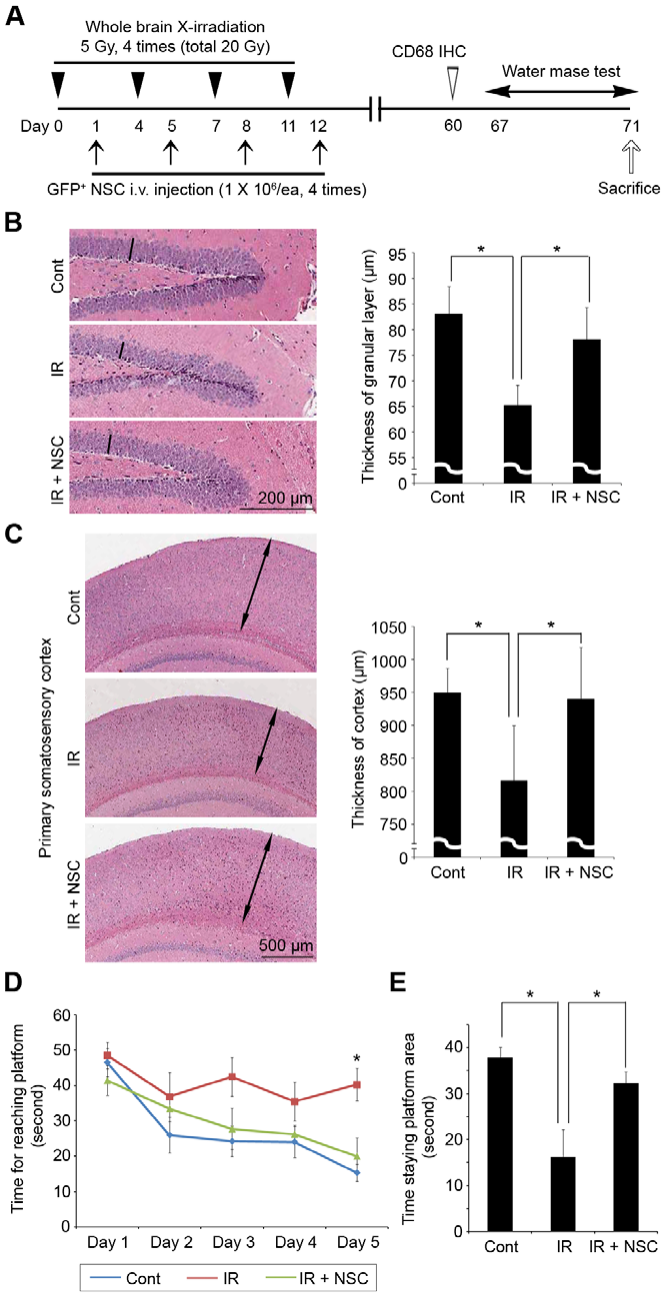
Effects of exogenous NSC supplementation on the brain structure and function after whole brain irradiation. (A) Experimental schedules. (B, C) At eight weeks after the last NSC supplementation, the depth of the granular layer of the dentate gyrus of the hippocampus (B) and the cerebral cortex (primary somatosensory cortex, C) were compared. The depth was measured at three random positions in a section (3 sections for each animal). Bars (B) or arrows (C) represent examples of the measurements. Cont  =  no irradiation with PBS injection (n = 7), IR  =  irradiation with PBS injection (n = 7). IR + NSC  =  irradiation with NSC supplementation (n = 6). * P<0.05. (D) Short term memory alteration was tested by measuring the length of time to locate the designated platform in the water bath everyday for five days (water maze test). * P<0.05. (E) The platform was removed and the time that the mice stayed at the platform area in one minute was measured and compared at Day 5 (probe trial). * P<0.05.

To make animal models representing the radiation-induced brain damage, 4×5 Gy whole brain X-irradiations (total 20 Gy, Day 0, 4, 7, and 11) were applied to C57BL/6 mice (Fig. 1A) using a device shielding the rest of the animal body from irradiation. The chronic inflammatory response of microglia after brain irradiation [19] was observed in our animal models by immunohistochemistry against CD68 (Day 60, n = 5, Fig. S2), confirming the effects of irradiation on the brain. CD68-positive cells were observed throughout the irradiated brains. To replenish the irradiated brains with NSCs, 1×10^6^ GFP^+^ NSCs in 100 µl PBS were systemically administrated (tail vein injection) at 24 hours after each irradiation (Cont, no irradiation with PBS injection, n = 7; IR, irradiation with PBS injection, n = 7; IR + NSC, irradiation with NSC supplementation, n = 6, Fig. 1A).

Eight weeks after the final NSC supplementation, the Morris water maze test [20] was performed on the Cont, IR, and IR + NSC groups to analyze short term memory change (Day 67–71), and corresponding brain structural alterations were examined (Day 71, Fig. 1A). The IR group mice had significantly reduced depths of both the granular layer of the dentate gyrus of the hippocampus and the cerebral cortex than the control group mice [65.2±4.0 µm vs 83.0±5.5 µm (Fig. 1B) and 815.9±83.7 µm vs 949.0±38.0 µm (Fig. 1C), respectively; P<0.05]. They also showed significantly poorer short term spatial memory acclimation, indicated by their inability to shorten their latency locating the designated platform even at Day 5 [Fig. 1D; 48.5±9.2 seconds (sec) in Day 1 and 40.3±11.1 sec Day 5; compare with Cont's 46.5±6.3 sec in Day 1 and 15.3±10.6 sec in Day 5]. In addition, they responded poorly to the probe trial (Day 5), indicated by the significantly shorter time of staying at the platform area after platform removal [Fig. 1E; 16.3±14.5 sec in one minute (min) vs 37.8±6.1 sec in one min; P<0.05]. In contrast, the mice with exogenous NSC supplementation had comparable depths of granular layer of the dendate gyrus of the hippocampus (Fig. 1B, 78.1±6.3 µm; P = 0.22) and the cerebral cortex (Fig. 1C, 939.4±79.8 µm; P = 0.83) with the control, with comparable latency in finding the platform (Fig. 1D, 41.4±11.0 sec in Day 1 and 20.0±13.8 sec in Day 5) and time of stay at the platform area (Fig. 1E, 32.3±6.5 sec in one min). Taken together, these data suggest that the exogenous NSC supplementation preserved both the brain structure and function after the whole brain irradiation.

### 3. Neuro-protective mechanisms of supplemented NSCs in the irradiated brains

**Figure 2:**
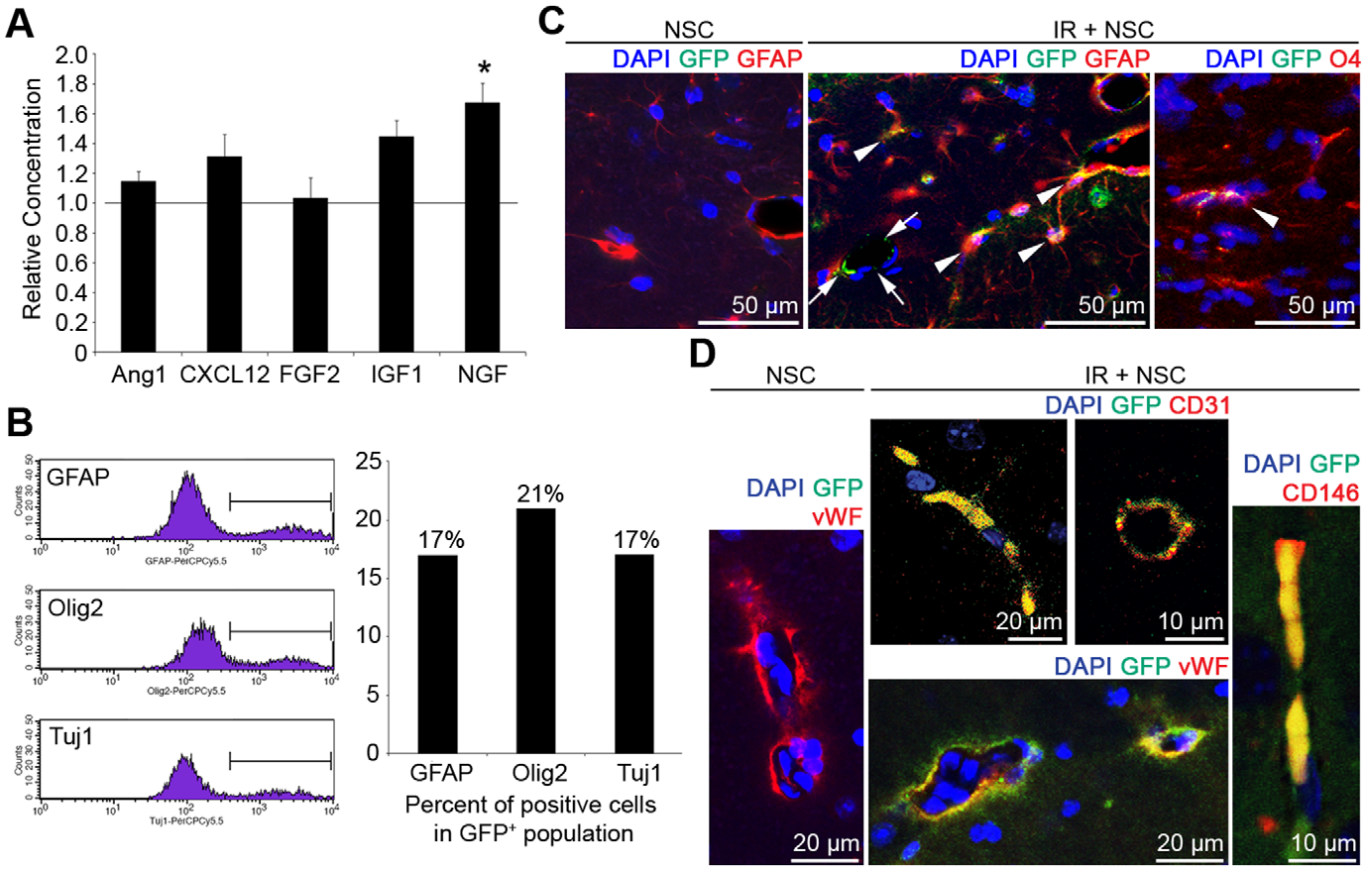
Neuro-protective mechanisms of NSCs against the radiation-induced brain damage. (A) Levels of Ang1, CXCL12, FGF2, IGF1 and NGF were compared between the IR and IR + NSC group by real-time PCR. Reference bar (1.0)  =  IR group. * P<0.05. (B) To determine the fate of injected GFP^+^ NSCs, brains of the IR + NSC mice were dissociated into single cells at 72 hours after the injection and expression of GFAP, Olig2, and Tuj1of GFP^+^ cells was analyzed by flow cytometry. (C, D) Brain sections were prepared from the IR + NSC or NSC mice, and immunohistochemistry against GFP (green) and GFAP, O4, CD31, vWF, or CD146 (red) was performed. Blue  =  nuclei.

Supplemented NSCs could make their neuro-protective effects by either secreting various cytokines (paracrine effects) or replacing damaged neural cells (replacement effects) [16]. Specific effects of NSC supplementation could be deviated in the animals with multiple injections of NSCs (Fig. 1A). Therefore, to find out therapeutic mechanisms of NSCs, PBS or 1×10^6^ NSCs were systemically injected to the mice that had one time 5 Gy whole brain irradiation at 24 hours before the injection (IR and IR + NSC, n = 5 for each group). 1×10^6^ NSCs were also systemically injected to the mice that did not have whole brain irradiation (NSC group, n = 5). When several neurotrophic factors such as Ang1, CXCL12, FGF2, IGF1, and NGF were compared between the IR and IR + NSC groups by quantitative PCR at 72 hours after the NSC injection, NGF was significantly increased in the brains of the IR + NSC group (Fig. 2A, right hemispheres of the mice were utilized, P<0.05), indicating paracrine effects. To determine the fate of injected GFP^+^ NSCs, the brains of the IR + NSC group were dissociated into single cells, and the expression of differentiated neural cell markers (GFAP, Olig2 and Tuj1) of GFP^+^ cells were analyzed by flow cytometry [left hemispheres (rostral half) was utilized]. Seventeen, 21, and 17% of GFP^+^ cells of the IR + NSC group expressed GFAP, Olig2, and Tuj1, respectively (Fig. 2B), indicating *in vivo* multi-potent differentiating potential of NSCs. GFP^+^ cells showed similar GFAP, Olig2, or Tuj1 positivity in the five IR + NSC mice. Co-localization of GFAP, O4 (for oligodencrocytes), or Tuj1 with GFP was also confirmed immunohistochemically in the brains of the IR + NSC mice [arrow heads in Fig. 2C; left hemispheres (caudal half) of the IR + NSC mice were utilized]. In contrast, few GFP^+^ cells were observed in the brains of the NSC group (Fig. 2C). Therefore, neuro-protective effects of supplemented NSCs in the irradiated brains could be mediated by both paracrine and replacement effects.

### 4. Trans-differentiation of NSCs into brain endothelial cells

While supplemented GFP^+^ NSCs were differentiated into astrocytes, oligodendrocytes, or neurons in the irradiated brains, GFP^+^ vessel-like structures were also observed (arrows, Fig. 2C). Interestingly, these tube-like structures did not co-express GFAP (Fig. 2C), indicating that they did not originate from GFP^+^ perivascular astrocytes. Immunohistochemistry against specific endothelial markers such as CD31, von Willebrand factor (vWF), and CD146 (Fig. 2D) confirmed that anti-GFP immunoreactivities co-localized with the specific markers of endothelial cells. In contrast, co-localization of GFP with CD31, vWF, or CD146 was not identified in the NSC group (Fig. 2D). Therefore, injected NSCs trans-differentiated into brain endothelial cells in the irradiated brains. It is possible that endothelial cells and/or endothelial progenitor cells (EPCs) might have contaminated the primarily cultured NSCs. However, we found few cells positive for endothelial (CD31) or endothelial progenitor cell markers (CD34 and Sca-1) among our primarily cultured NSCs (Fig. S3), excluding such possibility. CD31 expression was observed in 11% of GFP^+^ cells in the brains of the IR + NSC mice by flow cytometry (data not shown).

**Figure 3:**
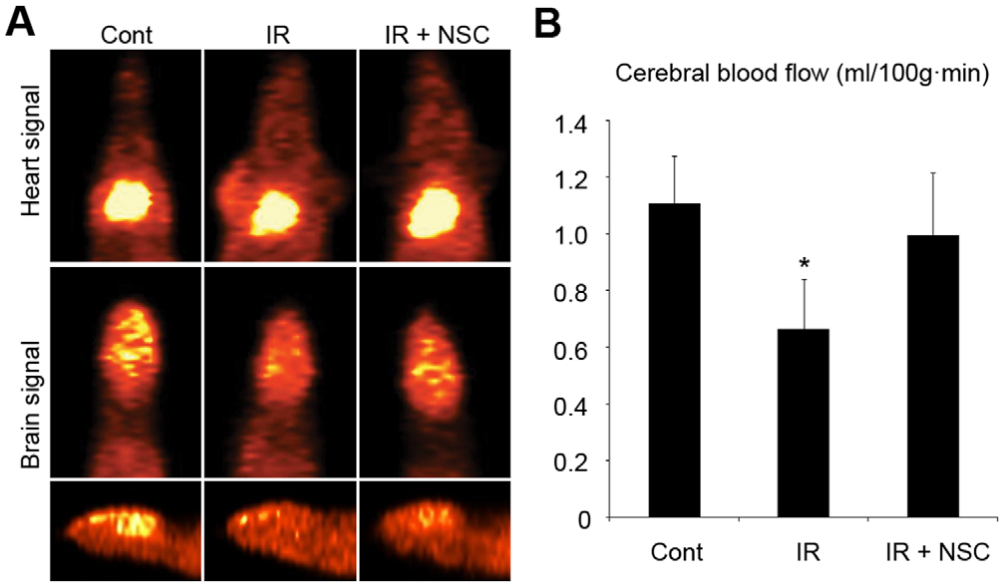
Restoration of the cerebral blood flow by supplementation of NSCs. 5 Gy whole brain irradiation was applied to mice, and NSCs were systemically supplemented at 24 hours after the irradiation. (A) H_2_^15^O was injected into the tail veins of the mice at 72 hours after the injection, and the injected tracer distributions in the brain and the heart were measured by H_2_^15^O PET imaging. (B) The cerebral blood flow (ml/100 g min) was determined by analyzing heart and brain signal. Cont, n = 3; IR, n = 5; IR + NSC, n = 3. Height  =  average. Error bar  =  standard deviation. * P<0.05.

**Figure 4:**
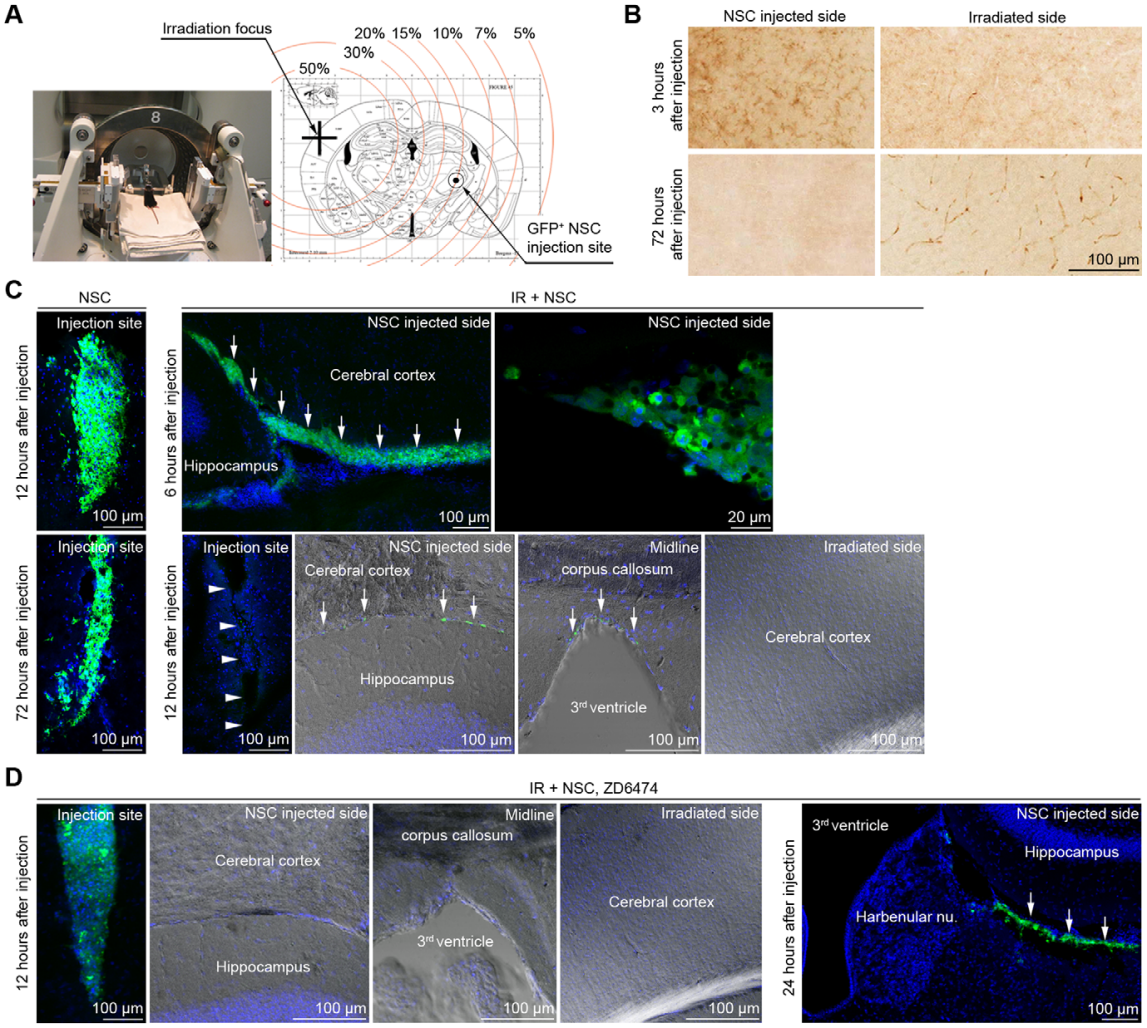
Trans-differentiation and migration of NSCs in the animal models with focal brain irradiation and effects of the VEGF signaling on the migration of NSCs. (A) GFP^+^ NSCs were injected into the right brain hemisphere stereotactically at 24 hours after focused brain irradiation to the left hemisphere using a GKS apparatus (50% isodose = 3 Gy). The dosimetry for GKS was calculated and presented. The irradiation focus could be within 0.5 mm of the planned target. (B) Immunohistochemistry against GFP was performed. Injected GFP^+^ NSCs migrated into the contralateral irradiated brain hemisphere and made GFP^+^ vessels. (C) The migration route and velocity were analyzed by immunohistochemistry against GFP (green) at various time points after the injection. NSC  =  NSC supplementation without GKS (n = 5 for each time point), IR + NSC  =  NSC supplementation at 24 hours after GKS (n = 5 for each time point). Arrowheads  =  injection tract. Arrows  =  GFP^+^ NSCs migrating in the subventricular zone. (D) Systemic treatment with a specific Flk-1 inhibitor (ZD6474, 50 mg/kg, oral administration 12 hours after the irradiation) inhibited the migration of injected GFP^+^ NSCs in the focally irradiated brain (n = 5 for each time point). Arrows  =  GFP^+^ NSCs migrating in the subventricular zone.

Next, we examined the effects of the whole brain irradiation and the supplementation of NSCs on the cerebral blood flow to observe the functional consequences of the trans-differentiation of NSCs. Positron emission tomography (PET) provides an excellent *in vivo* imaging technique for cerebral blood flow measurements [21], [22]. In the PET imaging, ^15^O labeled water (H_2_^15^O) was utilized as a tracer. The injected tracer distributions in the brain and the heart were measured *in vivo* as a function of time, and the cerebral blood flow was calculated with an arterial input function [23]. The H_2_^15^O PET imaging showed that the cerebral blood flow was reduced significantly at 96 hours after 5 Gy whole brain irradiation (Fig. 3, Cont = 1.11±0.17 ml/100 g min, n = 3; IR = 0.67±0.17 ml/100 g min, n = 5, P<0.05), and the reduction was reversed when 1×10^6^ NSCs were systemically supplemented at 24 hours after the irradiation (Fig. 4, 0.99±0.22 ml/100 g min, n = 3). These data demonstrated that endothelial cells differentiated from NSCs could take part in the restoration of the cerebral blood flow reduced by the whole brain irradiation.

### 5. Trans-differentiation and migration of NSCs in the animal models with focal brain irradiation

Although WBRT is widely used to treat brain metastases, the use of focal brain radiotherapy such as 3DCRT, IMRT, or radiosurgery, is more popular and increasing. To confirm the trans-differentiation of NSCs in the brains of animals with focal brain radiotherapy, we applied focal irradiation to the right cerebral cortex of C57BL/6 mouse using a GKS apparatus which can concentrate 50% of the maximal irradiation dose (50% isodose) within 1 mm (Fig. 4A, Fig. S5). The migration of NSCs to irradiated brain regions could be additionally analyzed in the animal models with GKS, since NSCs could be transplanted into the brain tissues of the opposite hemisphere that had low exposure to the radiation. Therefore, 1×10^5^ GFP^+^ NSCs in 10 µl HBSS were stereotactically injected into the opposite brain hemisphere at 24 hours after the focused irradiation (Fig. 4A). When GKS was not applied, injected NSCs remained in the injected site up to 72 hours (NSC group, Fig. 4C, n = 5 for each time point). In the IR + NSC group (n = 5 for each time point), NSCs left the injection site (arrowheads, Fig. 4C), migrated along the subventricular zone (arrows, Fig. 4C), crossed the midline in the subventricular zone beneath the corpus callosum at 12 hours after the injection (arrows in Fig. 4C), and formed GFP^+^ microvessels in the irradiated hemisphere (immunohistochemistry against GFP, Fig. 4B).

### 6. Involvement of the VEGF signaling in the migration and trans-differentiation of NSCs

VEGF is a critical force that drives adult vasculogenesis and one of the most potent molecules triggering an EPC release from the bone marrow [24]. We checked the expression of VEGF receptors on NSCs using semi-quantitative RT-PCR, immunocytochemistry, and flow cytometry. The expression of the VEGF receptor 2 (Flk-1) was prominent in NSCs (Fig. S4A–C). VEGF expression and secretion of NSCs were significantly increased by *in vitro* irradiation (Fig. S4D, E). In addition, levels of VEGF were significantly increased in the irradiated brains (5 and 10 Gy, n = 5 for each group) compared to the control brains (n = 5, Fig. S4F). Therefore, VEGF signaling could be hypothesized to have an important role in the endothelial cell repopulation process of NSCs.

At 24 hours after the focused irradiation (Fig. 4A), followed by a systemic administration of ZD6474, a specific Flk-1 inhibitor (Fig. S6, ZD6476 group, n = 5 for each time point) [25], 1×10^5^ GFP^+^ NSCs in 10 µl HBSS were stereotactically injected into the opposite brain hemisphere. Compared with the IR + NSC group, inhibition of the VEGF signaling made the injected NSCs to remain in the injection site until 12 hours after the injection (Fig. 4D). Even at 24 hours after the injection, NSCs could not cross the midline (arrows, Fig. 4D).

**Figure 5:**
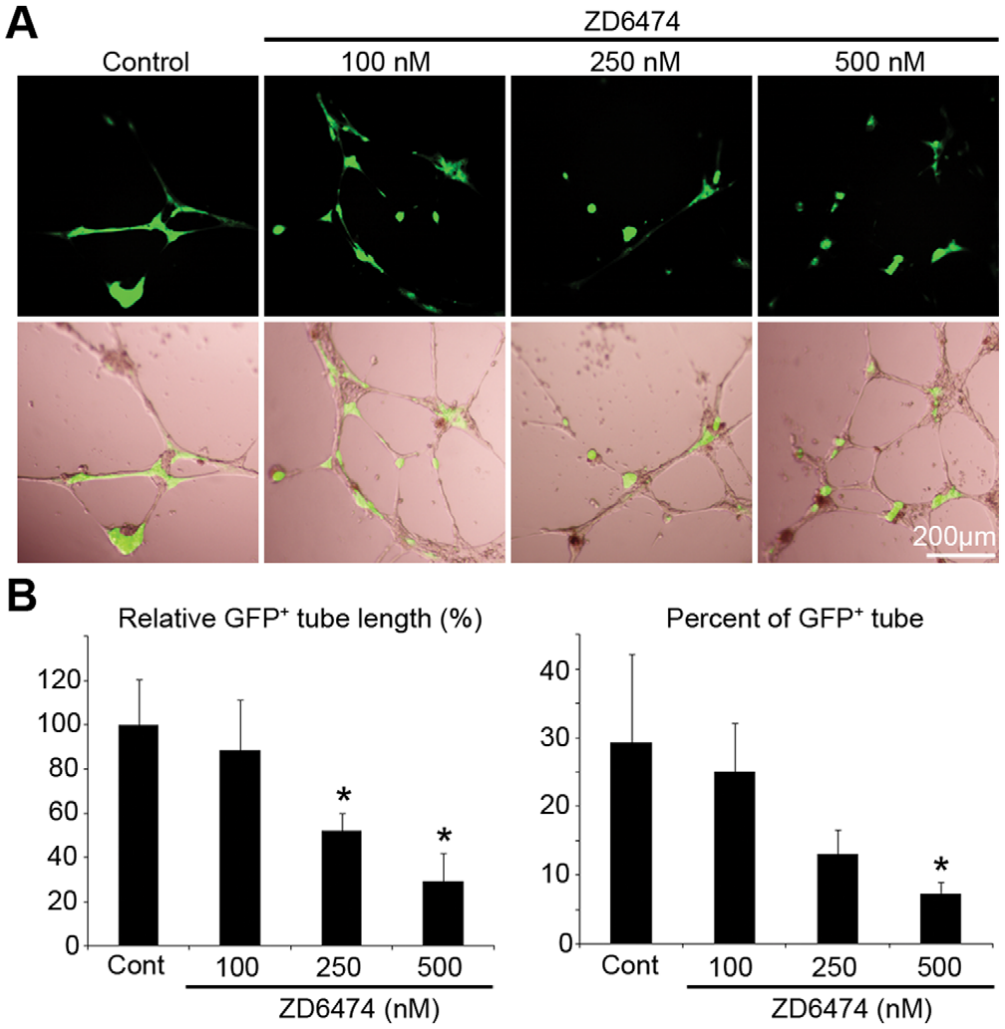
Effects of the VEGF signaling on the trans-differentiation of NSCs. (A) GFP-positive NSCs and GFP-negative human umbilical venous endothelial cells were co-cultured on the matrigel in the differentiation condition of endothelial cells. Effects of Flk-1 inhibition on the trans-differentiation of NSCs were analyzed by the treatment of ZD6474. (B) Relative GFP^+^ tube length (%) and percent of GFP^+^ tube of total tube length were measured and compared * P<0.05. n = 3 for each group.

The effects of Flk-1 inhibition on the trans-differentiation of NSCs were also analyzed by co-culturing GFP^+^ NSCs with human umbilical venous endothelial cells (HUVECs) on the matrigel [26]. HUVECs made numerous tubes in the differentiation condition, while NSCs remained as spheres (Fig. S7). When they were co-cultured, GFP^+^ tubes were made effectively (Fig. 5A). However, treatment of ZD6474 specifically and dose-dependently inhibited the formation of GFP^+^ tube and made GFP^+^ NSCs to remain as spheres (Fig. 5B, P<0.05). Morphologically, the viability of NSCs on the matrigel was not affected by the treatment of ZD6474, thus excluding the possibility that the reduction of GFP^+^ tube formation originates from toxic effects. These data suggest that signaling through the Flk-1 is important in both the migration and trans-differentiation of NSCs.

## Discussion

In this study, we provide *in vivo* translation data indicating that exogenous NSC supplementation can be a novel preventive and/or therapeutic modality against the radiation-induced brain damage. Most radiation-induced brain damage originates from medical treatments, yet the unfortunate reality is that the frequency of radiation therapy for the brain is expected to further increase in the future. Therefore successful combination of brain radiation therapy and NSC supplementation would provide a highly promising therapeutic option for patients with brain diseases. In addition, accidental exposure to large doses of irradiation could result in neurological effects and rapid death [27]. Exogenous NSC supplementation could also be considered in these cases with other supportive cares. Previously, neuro-protective effects of NSCs were reported to be mediated by the secretion of various neurotrophic factors (paracrine effects) and/or the replacement of damaged neural cells (replacement effects) [16]. In our animal models with radiation-induced brain damage, NGF was significantly increased in the brain by the supplementation of NSCs. In addition, transplanted NSCs differentiated into various neural cells such as neurons, astrocytes and oligodentrocytes in the irradiated brains. These results suggest that both the paracrine and replacement effects were implicated in the preventive and/or therapeutic outcome of NSCs against radiation-induced brain damage.

Interestingly, NSCs supplemented into irradiated brains trans-differentiated into brain endothelial cells. The reduction of the cerebral blood flow by irradiation was reversed by NSC supplementation. Therefore, the trans-differentiation into endothelial cells would be one of the preventive and/or therapeutic mechanisms of NSCs against radiation-induced brain damage. In the resting state, the brain receives 15–20% of the body's blood flow since it is one of the most metabolically active organs [28]. Moreover, the brain is highly dependent on the sufficient blood supply for its survival and functions [29]. One microvessel supports numerous neural cells; therefore, the replacement of damaged endothelial cells could be much more important than the regeneration of lost neural cells for the maintenance of brain structures and functions. Since the report about the trans-differentiation potential of NSCs into endothelial cells [26], its physiological and clinical implications have remained to be elucidated. Recently, it was reported that glioblastoma stem-like cells, which could originate from NSCs and maintain the key molecular properties of NSCs, show endothelial differentiation *in vitro* and *in vivo* [30], [31]. Here, we also demonstrated that NSCs indeed replace damaged endothelial cells in pathological conditions to prevent structural and functional alterations of the brain. More importantly, the replacement activity was functionally relevant, evidenced by the restoration of the cerebral blood flow.

Active vascularization induces and supports the development of the brain [32]. Therefore, EPCs and/or differentiated endothelial cells could be included in the NSCs primarily cultured from brains of 13.5 day old mouse embryos. To exclude this possibility, we examined the CD31, CD34 and Sca-1 expression of cultured NSCs, but there were few CD31, CD34, and Sca-1-positive cells. In addition, NSCs could not make tubes on matrigels in the tube formation assay, whereas endothelial cells could. This indicates that cultured NSCs contain, if any, few EPCs or endothelial cells. VEGF is a critical force that drives vasculogenesis and one of the most potent molecules triggering EPC release from the bone marrow [24]. We demonstrated that the VEGF signaling is also essential in the migration and trans-differentiation of NSCs, which suggests that VEGF would be a common mediator that recruits stem cells for the repair of damaged vessels.

In the brain parenchyma, NSCs are closely related with their niche, of which the endothelial cell is one of the most essential components [32]–[34]. Traditionally the niche has been reported to regulate the self-renewal and fate of NSCs using various kinds of signaling pathways [32]–[34]. However our data provoked the possibility that NSCs might reciprocally have the potential to reconstruct their niche by themselves. This would further support the self-renewal and regeneration potential of NSCs, although the exact functional implications still need to be elucidated.

*In vitro* long-term expansion capacity [17] and extensive functional stability and plasticity [12]–[14] of NSCs have provoked the possibility of regenerative medicine for various neurological diseases, such as stroke, spinal cord injury and neurodegenerative diseases, which currently have few effective therapeutic modalities [35]. The survival of injected NSCs could be increased when endothelial cells derived from NSCs would secrete various survival factors for NSCs. In addition, therapeutic effect could be potentiated when damaged endothelial cells and neural cells could be replaced simultaneously by NSCs, because endothelial cells are also affected by those diseases [36]. Therefore, novel therapeutic methods which utilize the trans-differentiation capacity of NSCs would maximize the therapeutic effects of regenerative technologies using NSCs.

In summary, our data demonstrate that exogenous NSC supplementation could recover radiation-induced functional losses of the brain. Although the brain is sensitive to radiation, radiation therapy remains as an indispensable therapeutic modality for various brain diseases. Therefore, successful combination of the brain radiation therapy and NSC supplementation would provide a highly promising therapeutic option for patients with brain diseases.

## Materials and Methods

### 1. Primary culture of mouse fetal GFP^+^ NSCs

All animal experiments were approved by the appropriate Institutional Review Boards of the Samsung Medical Center (Seoul, Korea, approval ID  =  C-A7-220-3) and conducted in accord with the ‘National Institute of Health Guide for the Care and Use of Laboratory Animals’ (NIH publication No. 80-23, revised in 1996). Brains obtained from 13.5 day old GFP transgenic C57BL/6 mouse embryos (C57BL/6-Tg(UBC-GFP)30Scha/J, The Jackson Laboratory) [18] were mechanically dissociated into single cells. Dissociated cells were cultured in the NeuroBasal Media (Invitrogen) supplemented with N2, B27 (Invitrogen) and recombinant EGF (50 ng/ml; R&D Systems). To differentiate cultured cells, cells were plated onto poly-L-lysine (PLO)-precoated culture dish (Invitrogen) and were subject to growth in DMEM with 10% fetal bovine serum (10% FBS/DMEM; Cambrex).

### 2. Immunocytochemistry

Immunocytochemistry was performed using a standard method. Briefly, cells were fixed in ice-cold methanol/acetone (1∶1) for 10 minutes. After incubation in 0.2% Triton X-100/PBS cells were treated with primary antibodies; Sox2 (R&D Systems), Nestin, Musashi, CD133, Tuj1, GFAP, Olig2 (Millipore), and Flk-1 (Cell Signaling Technology).

### 3. Whole brain irradiation and systemic supplementation of NSCs

Four serial whole brain X-irradiations, each with 5 Gy, were applied to C57BL/6 mice using a blood irradiator (IBL-437C, CIS-US, Inc.). Mice bodies were shielded with a custom-made lead shielding device. At 24 hours after each irradiation, 1×10^6^ GFP^+^ NSCs in 100 µl PBS were administrated into the tail vein.

### 4. Morris water maze test

Mice were trained on Morris water maze [20], three trials per day for five days. At the start of each trial, one mouse was gently placed into the water with its head facing the outside of the tank. The start location was randomized, while ensuring no location was used repeatedly in consecutive trials. A total of eight start locations were used for the test, which were evenly spaced around the maze. SMART video-tracking system (Panlab s.l.u.) was used to measure the length of time to locate the designated platform in the water bath. A trial lasted either 60 sec or until the mouse reached the platform and remained on the platform for 10 sec. If a mouse did not reach the platform within 60 sec, it was gently guided there by hand. Mice were placed back in their cage and allowed to rest for 30 sec between trials. One hour after training on Day 5, the platform was removed and mice were tested with a probe trial; the time that the mice stayed at the platform area in one minute was measured.

### 5. Specimens and immunohistochemistry

The animals were anesthetized, and perfusion fixation was performed by transcardially perfusing 4°C cold PBS containing heparin (1 IU/ml), followed by ice-cold 4% paraformaldehyde in PBS. The brains were immediately removed, postfixed in the same fixative overnight and processed for paraffin and frozen embedding using standard experimental procedure. H&E staining was performed for paraffin sections, and the thickness of the granular layer of the dentate gyrus of the hippocampus and the cerebral cortex (primary somatosensory cortex) was measured using a slide scanner (ScanScope® CS, Aperio Technologies, Inc.) and an image analyze software (ImageScope, Aperio Technologies, Inc.). The thickness was measured at three random positions in a section, and three sections were analyzed for each animal. Mean thickness was calculated for each animal and compared. The frozen blocks were cut into 40 µm coronal sections. Immunohistochemistry was performed with the free-floating method using the following antibodies: CD68, von Willebrand factor (Abcam), GFP (Invitrogen), Tuj1, GFAP, O4, CD146 (Millipore) and CD31 (BD Pharmingen).

### 6. Quantitative RT-PCR

Total RNAs were isolated from mouse brains, GFP^+^ NSCs, and HUVECs using Total RNA Purification Kit (Qiagen) according to the manufacturer's protocol. Equal amounts of RNA were subjected to cDNA synthesis by using Superscript™ III First-Strand Synthesis System (Invitrogen). One microliter of the first-strand cDNA reaction mixture and 0.2 µM of each primer set [37] were used for semi-quantitative RT-PCR (35 cycles for VEGF receptors, 18 cycles for GAPDH). In real-time quantitative PCR, relative amount of each mRNA was evaluated by Roche LC480 real-time quantitative PCR detection system (Roche) and calculated following normalization to the GAPDH mRNA. The primer sequences were described in Table S1.

### 7. Flow cytometry

Primary antibodies against Sox2 (R&D Systems), Musashi, Olig2, Tuj1 (Milipore), and GFAP (Santa Cruz biotechnology) were conjugated with PerCP-Cy5.5 using a conjugation kit (AbD Serotec). PerCP-Cy5.5 conjugated anti-Nestin, Flk-1 (BD Pharmingen), and Sca-1 (eBioscience) antibody and PE conjugated anti-CD31 and CD34 antibody (BD Pharmingen) were utilized in the flow cytometry. Brains were enzymatically dissociated into single cells, and red blood cells were removed by differential centrifugation. Dissociated cells were fixed and permeabilized by the Cytofix/Cytoperm™ kit (BD Biosciences), labeled with antibodies, and then analyzed by a FACS Calibur machine (BD Biosciences).

### 8. H_2_^15^O positron emission tomography (PET) imaging

The PET experiments were performed according to methods reported previously [23]. An Inveon™ preclinical small animal PET system (Siemens Medical Solutions), which allows simultaneous imaging of brains and hearts of two mice in one scanner field of view, was used. Anesthetized animals were moved to the scanner bed and placed in supine position. The ^15^O labeled tracers (H_2_^15^O) were produced by an on-site PETtrace cyclotron (GE Healthcare) and delivered to the PET room for each PET scan. A dynamic PET scan (24×5 sec frames) was started with the simultaneous initiation of H_2_^15^O administration (bolus injection into tail veins, 1 mCi in 0.2 ml saline). The cerebral blood flow was calculated with an arterial input function by numerically solving the equations reported previously [23]. The brain regions of interest were defined as regions with elevated signals in the control mice (yellow in Fig. 3A). The signals for the same regions of the IR and IR + NSC mice were analyzed.

### 9. Focused brain irradiation and orthotopic NSC injection

Focused brain irradiation was applied to the right cerebral cortex of C57BL/6 mouse using a gamma knife surgery apparatus (Leksell Gamma Knife® 4C, Elekta AB). A stereotactic frame (Leksell frame) for the mouse was made (Fig. 4A), and the irradiation accuracy and concentrating capacity were tested using radiosensitive films (Fig. S5). At 24 hours after the focused irradiation, 1×10^5^ GFP^+^ NSCs suspended in 10 µl HBSS were stereotactically injected into the mouse brains (2 mm left and 1 mm anterior to the bregma, 2 mm deep).

### 10. *In vitro* trans-differentiation of NSCs

HUVECs (American Type Culture Collection) were cultured in endothelial cell growth medium (EGM) supplemented with EGM SingleQuots (EGM complete media, Lonza). BD BioCoat™ Angiogenesis System (BD Biosciences) was used for the tube formation test. 2×10^4^ HUVECs, 2×10^4^ GFP^+^ NSCs, or 1×10^4^ HUVECs + 1×10^4^ GFP^+^ NSCs in 500 µl EGM complete media supplemented with 10% FBS were cultured on matrigel in 96-well plates for 16 hours according to the manufacturer's protocol. 100, 250, or 500 nM of ZD6474 (LC Labs) was treated to the co-culture wells. Picture of each well was taken, and the sums of total tube length were calculated. In parallel, GFP^+^ tubes were quantified. Relative GFP^+^ tube lengths and percent of GFP^+^ tube (length of GFP^+^ tube/length of total tube) were calculated and compared.

### 11. Statistical analysis

Statistical comparisons between groups were performed using the Student's t-test. Values of P<0.05 were considered statistically significant.

## Supporting Information

Figure S1 Fetal mouse NSCs expressing GFP were primarily cultured from brains of 13.5 day old GFP transgenic C57BL/6 mouse embryos. Expression of NSC markers (Nestin, Musashi, Sox2, and CD133) and differentiated neural cell markers (Tuj1 for the neuron; GFAP for the astrocyte; Olig2 for the oligodendrocyte) was examined by immunocytochemistry (**A**, **C**, **D**, **E**) or flow cytometry (**B**). NSCs forming neurospheres in the NSC culture condition without serum were utilized (**A**, **B**). NSCs maintained in 10% FBS/DMEM on PLO-coated slides for overnight (**C**), 4 days (**D**), and 2 weeks (**E**) were analyzed.
(DOC)

Figure S2 The chronic inflammatory response of microglia after the brain irradiation was observed in the irradiated mice by anti-CD68 immunohistochemistry at seven weeks after the last irradiation. There were few CD68-positive cells in the brains of the control mice. CD68-positive cells (arrowheads) were magnified in the insets.
(DOC)

Figure S3 Expression of endothelial or endothelial progenitor cell markers (CD31, CD34 and Sca-1) of primarily cultured GFP^+^ NSCs was analyzed by flow cytometry and compared with those of endothelial cells (bEND.3). Few primarily cultured GFP^+^ NSCs expressed the endothelial or endothelial progenitor cell markers.
(DOC)

Figure S4 Expression of VEGF receptors in NSCs was analyzed by semi-quantitative RT-PCR (A), immunocytochemistry (B), and flow cytometry (C). The VEGF receptor 2 (Flk-1) was predominantly expressed in NSCs while all VEGF receptors were highly expressed in endothelial cells. GAPDH  =  internal control. VEGF concentration in the culture medium (D) and VEGF expression of NSCs (E) were analyzed by ELISA and Real-Time PCR, respectively, at 24 hours after 0, 2, 4, or 8 Gy *in vitro* irradiation. n = 3 for each group. * P<0.05. (F) Changes in VEGF concentration of the brain were examined by ELISA at 24 hours after 0, 5, or 10 Gy whole brain irradiation (n = 5 for each group). * P<0.05.
(DOC)

Figure S5 The gamma knife surgery device can concentrate 50% of the maximal irradiation dose (50% isodose) within 1 mm. The concentration capacity was tested using radiosensitive films. Blue spots represent the intensity of the irradiation and the intensity is presented as graphs.
(DOC)

Figure S6 Detailed experimental schedule to test the effects of KDR inhibition on the migration of NSCs is illustrated.
(DOC)

Figure S7 GFP-negative human umbilical venous endothelial cells (HUVECs) made numerous tubes in matrigels when given differentiation conditions, while GFP-positive NSCs remained as spheres.
(DOC)

Table S1 Oligonucleotide primers and probes used for mRNA expression analysis by real-time PCR.
(DOC)
