# Supplementary material for: Trans-Differentiation of Neural Stem Cells: A Therapeutic Mechanism Against the Radiation Induced Brain Damage
Source: PLoS One. 2012 Feb 10;7(2):e25936. doi: 10.1371/journal.pone.0025936 (PMC3277599; doi:10.1371/journal.pone.0025936)

**Figure S1.** Fetal mouse NSCs expressing GFP were primarily cultured from brains of 13.5 day old GFP transgenic C57BL/6 mouse embryos. Expression of NSC markers (Nestin, Musashi, Sox2, and CD133) and differentiated neural cell markers (Tuj1 for the neuron; GFAP for the astrocyte; Olig2 for the oligodendrocyte) was examined by immunocytochemistry (**A**, **C**, **D**, **E**) or flow cytometry (**B**). NSCs forming neurospheres in the NSC culture condition without serum were utilized (**A**, **B**). NSCs maintained in 10% FBS/DMEM on PLO-coated slides for overnight (**C**), 4 days (**D**), and 2 weeks (**E**) were analyzed.


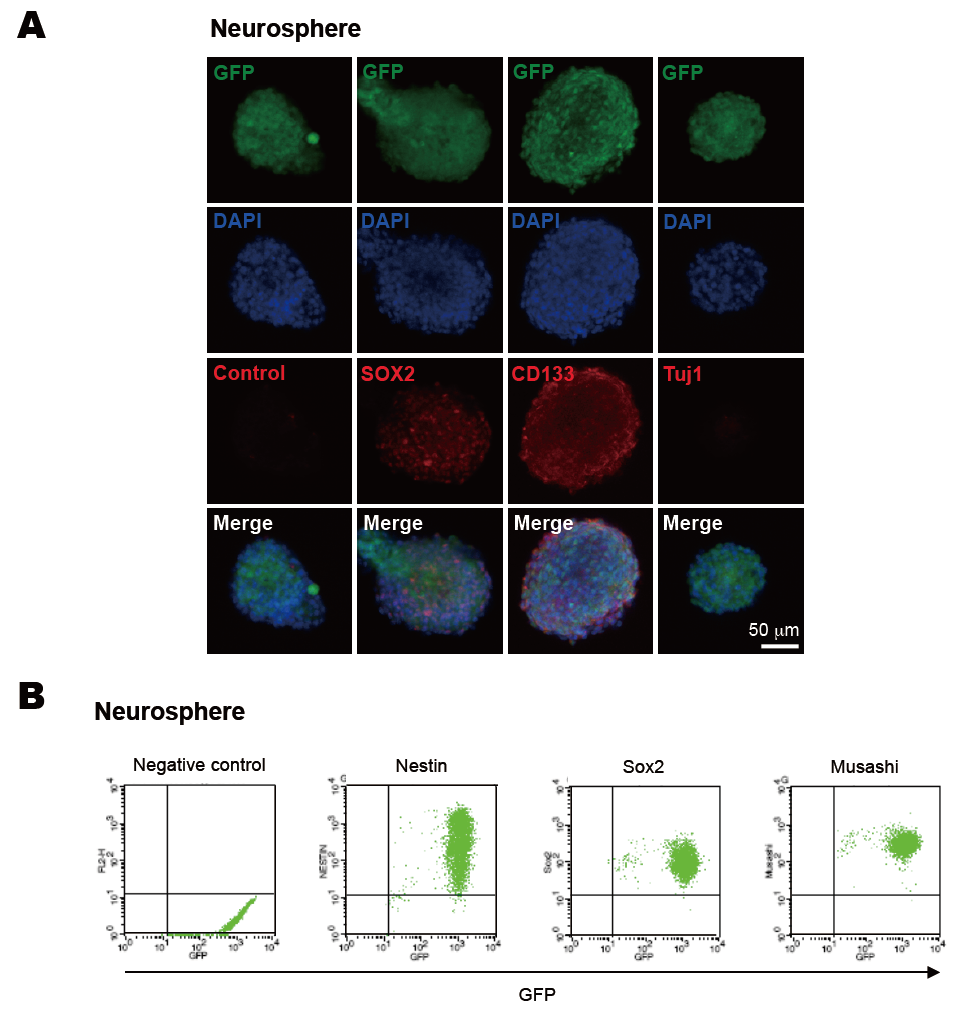


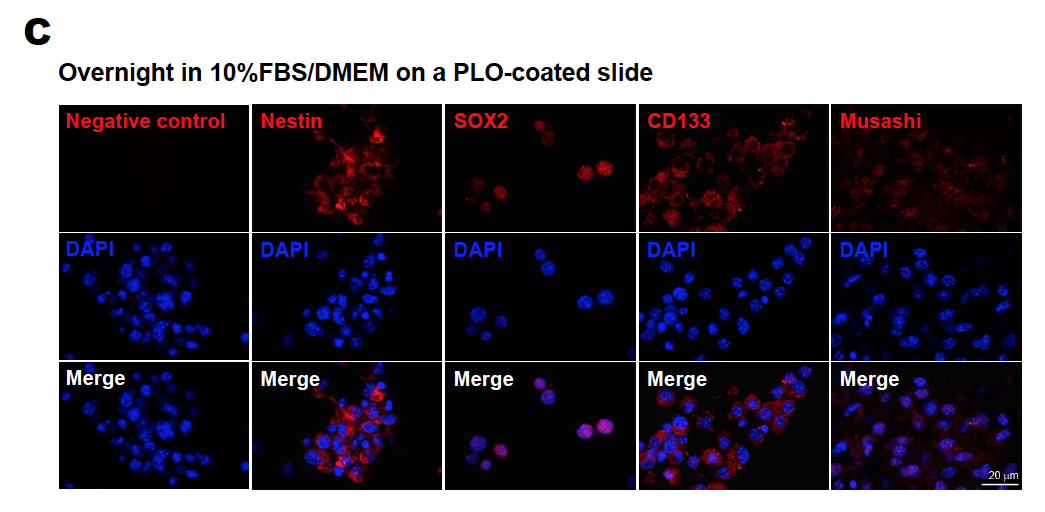


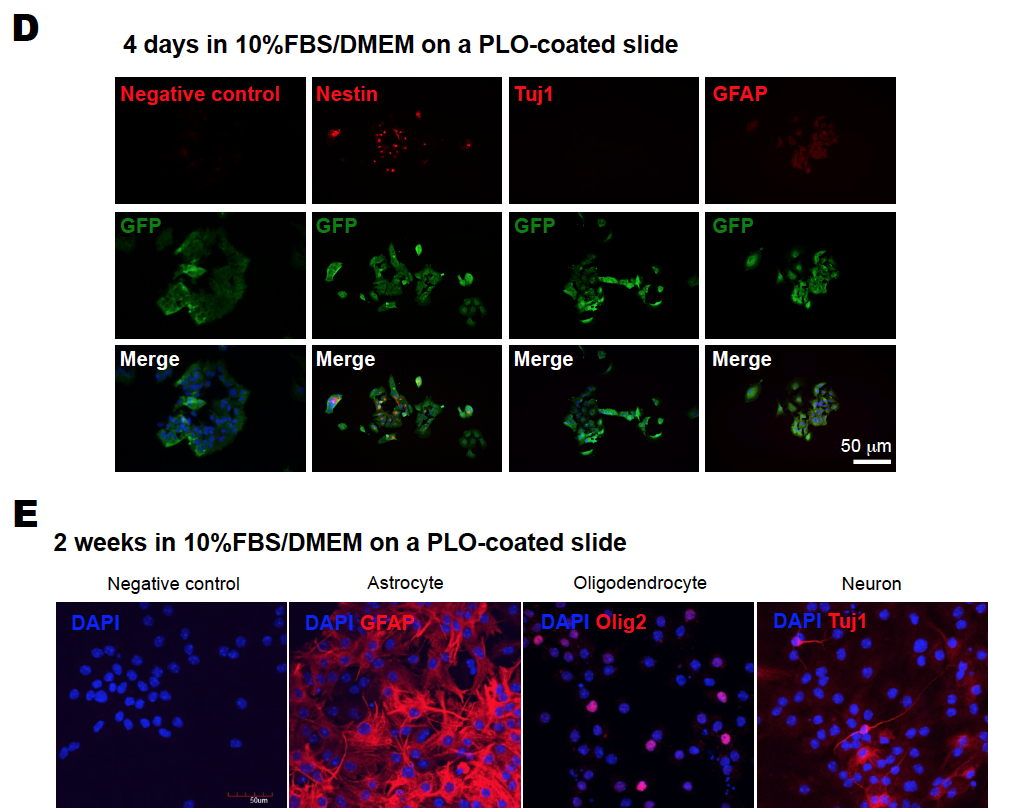

Supplement: Figure S1 — Fetal mouse NSCs expressing GFP were primarily cultured from brains of 13.5 day old GFP transgenic C57BL/6 mouse embryos. Expression of NSC markers (Nestin, Musashi, Sox2, and CD133) and differentiated neural cell markers (Tuj1 for the neuron; GFAP for the astrocyte; Olig2 for the oligodendrocyte) was examined by immunocytochemistry (A, C, D, E) or flow cytometry (B). NSCs forming neurospheres in the NSC culture condition without serum were utilized (A, B). NSCs maintained in 10% FBS/DMEM on PLO-coated slides for overnight (C), 4 days (D), and 2 weeks (E) were analyzed. (DOC) [file pone.0025936.s001.doc]
