# Supplementary material for: Trans-Differentiation of Neural Stem Cells: A Therapeutic Mechanism Against the Radiation Induced Brain Damage
Source: PLoS One. 2012 Feb 10;7(2):e25936. doi: 10.1371/journal.pone.0025936 (PMC3277599; doi:10.1371/journal.pone.0025936)

**Figure S2.** The chronic inflammatory response of microglia after the brain irradiation was observed in the irradiated mice by anti-CD68 immunohistochemistry at seven weeks after the last irradiation. There were few CD68-positive cells in the brains of the control mice. CD68-positive cells (arrowheads) were magnified in the insets.


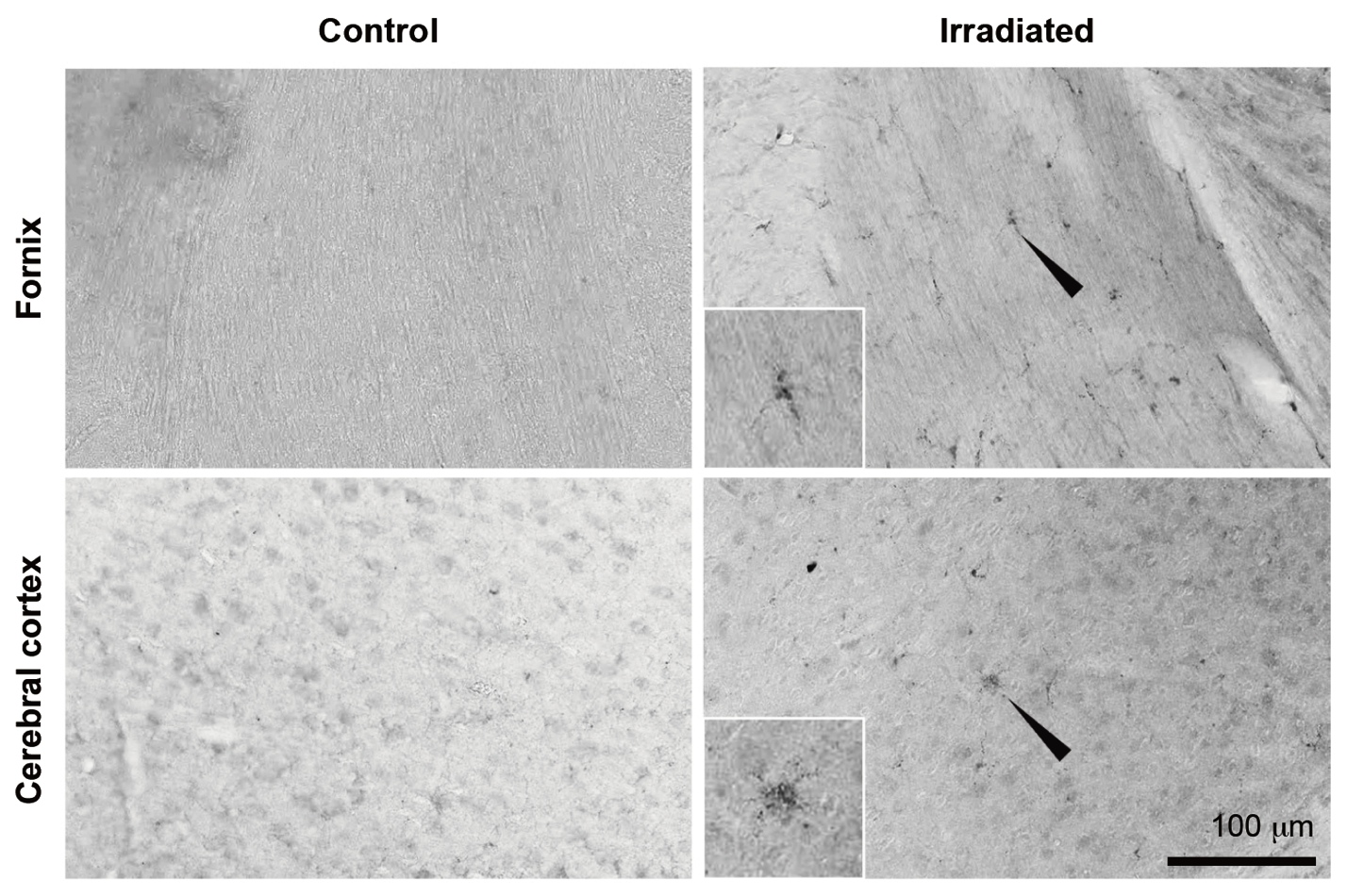

Supplement: Figure S2 — The chronic inflammatory response of microglia after the brain irradiation was observed in the irradiated mice by anti-CD68 immunohistochemistry at seven weeks after the last irradiation. There were few CD68-positive cells in the brains of the control mice. CD68-positive cells (arrowheads) were magnified in the insets. (DOC) [file pone.0025936.s002.doc]
