# Supplementary material for: Trans-Differentiation of Neural Stem Cells: A Therapeutic Mechanism Against the Radiation Induced Brain Damage
Source: PLoS One. 2012 Feb 10;7(2):e25936. doi: 10.1371/journal.pone.0025936 (PMC3277599; doi:10.1371/journal.pone.0025936)
Supplement: Figure S3 — Expression of endothelial or endothelial progenitor cell markers (CD31, CD34 and Sca-1) of primarily cultured GFP+ NSCs was analyzed by flow cytometry and compared with those of endothelial cells (bEND.3). Few primarily cultured GFP+ NSCs expressed the endothelial or endothelial progenitor cell markers. (DOC) [file pone.0025936.s003.doc]

**Figure S3.** Flow cytometric analysis of primarily cultured GFP^+^ NSCs. Expression of endothelial or endothelial progenitor cell markers (CD31, CD34 and Sca-1) of primarily cultured GFP^+^ NSCs was analyzed by flow cytometry and compared with those of endothelial cells (bEND.3). Few primarily cultured GFP^+^ NSCs expressed the endothelial or endothelial progenitor cell markers

**
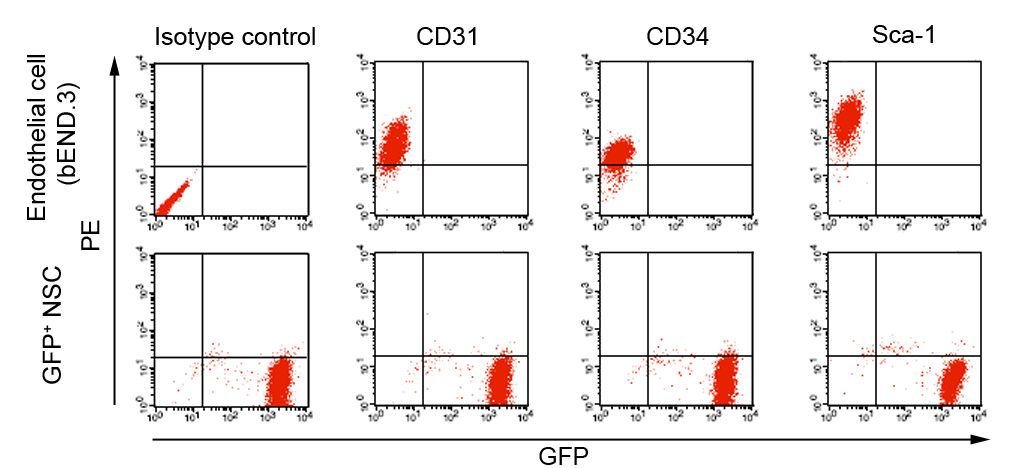
**
