# Supplementary material for: Trans-Differentiation of Neural Stem Cells: A Therapeutic Mechanism Against the Radiation Induced Brain Damage
Source: PLoS One. 2012 Feb 10;7(2):e25936. doi: 10.1371/journal.pone.0025936 (PMC3277599; doi:10.1371/journal.pone.0025936)

**Figure S4.** Expression of VEGF receptors in NSCs was analyzed by semi-quantitative RT-PCR (A), immunocytochemistry (B), and flow cytometry (C). The VEGF receptor 2 (Flk-1) was predominantly expressed in NSCs while all VEGF receptors were highly expressed in endothelial cells. GAPDH = internal control. VEGF concentration in the culture medium (D) and VEGF expression of NSCs (E) were analyzed by ELISA and Real-Time PCR, respectively, at 24 hours after 0, 2, 4, or 8 Gy *in vitro* irradiation. n = 3 for each group. * P < 0.05. (F) Changes in VEGF concentration of the brain were examined by ELISA at 24 hours after 0, 5, or 10 Gy whole brain irradiation (n = 5 for each group). * P < 0.05.


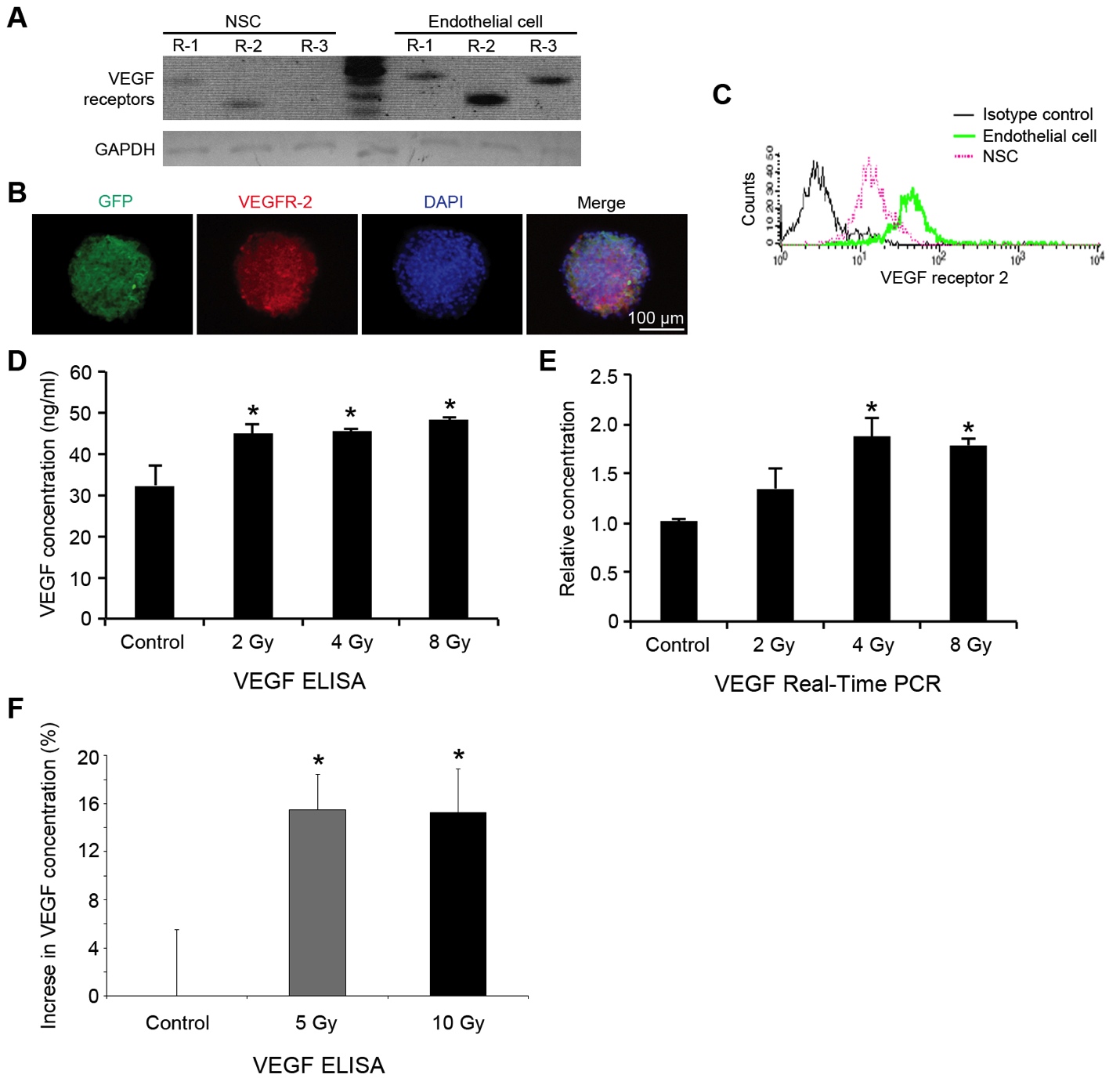

Supplement: Figure S4 — Expression of VEGF receptors in NSCs was analyzed by semi-quantitative RT-PCR (A), immunocytochemistry (B), and flow cytometry (C). The VEGF receptor 2 (Flk-1) was predominantly expressed in NSCs while all VEGF receptors were highly expressed in endothelial cells. GAPDH = internal control. VEGF concentration in the culture medium (D) and VEGF expression of NSCs (E) were analyzed by ELISA and Real-Time PCR, respectively, at 24 hours after 0, 2, 4, or 8 Gy in vitro irradiation. n = 3 for each group. * P<0.05. (F) Changes in VEGF concentration of the brain were examined by ELISA at 24 hours after 0, 5, or 10 Gy whole brain irradiation (n = 5 for each group). * P<0.05. (DOC) [file pone.0025936.s004.doc]
