# Supplementary material for: Trans-Differentiation of Neural Stem Cells: A Therapeutic Mechanism Against the Radiation Induced Brain Damage
Source: PLoS One. 2012 Feb 10;7(2):e25936. doi: 10.1371/journal.pone.0025936 (PMC3277599; doi:10.1371/journal.pone.0025936)

**Figure S5.** The gamma knife surgery device can concentrate 50% of the maximal irradiation dose (50% isodose) within 1 mm. The concentration capacity was tested using radiosensitive films. Blue spots represent the intensity of the irradiation and the intensity is presented as graphs.


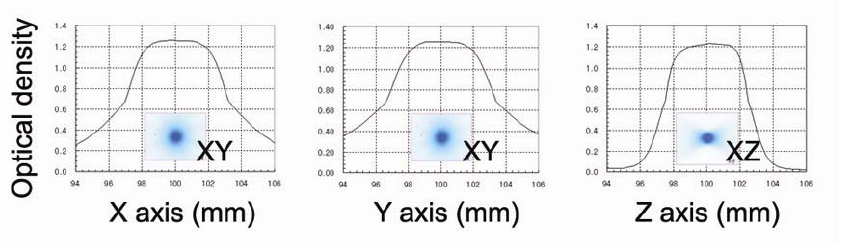

Supplement: Figure S5 — The gamma knife surgery device can concentrate 50% of the maximal irradiation dose (50% isodose) within 1 mm. The concentration capacity was tested using radiosensitive films. Blue spots represent the intensity of the irradiation and the intensity is presented as graphs. (DOC) [file pone.0025936.s005.doc]
