# Supplementary material for: Trans-Differentiation of Neural Stem Cells: A Therapeutic Mechanism Against the Radiation Induced Brain Damage
Source: PLoS One. 2012 Feb 10;7(2):e25936. doi: 10.1371/journal.pone.0025936 (PMC3277599; doi:10.1371/journal.pone.0025936)

**Figure S6.** Detailed experimental schedule to test the effects of KDR inhibition on the migration of NSCs.


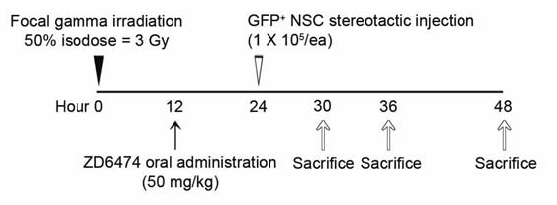

Supplement: Figure S6 — Detailed experimental schedule to test the effects of KDR inhibition on the migration of NSCs is illustrated. (DOC) [file pone.0025936.s006.doc]
