# Supplementary material for: Trans-Differentiation of Neural Stem Cells: A Therapeutic Mechanism Against the Radiation Induced Brain Damage
Source: PLoS One. 2012 Feb 10;7(2):e25936. doi: 10.1371/journal.pone.0025936 (PMC3277599; doi:10.1371/journal.pone.0025936)

**Figure S7.** GFP-negative human umbilical venous endothelial cells (HUVECs) made numerous tubes on matrigels when given differentiation conditions, while GFP-positive NSCs remained as spheres.


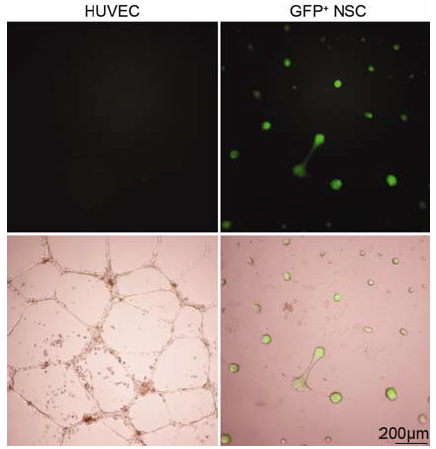

Supplement: Figure S7 — GFP-negative human umbilical venous endothelial cells (HUVECs) made numerous tubes in matrigels when given differentiation conditions, while GFP-positive NSCs remained as spheres. (DOC) [file pone.0025936.s007.doc]
