# Supplementary material for: Trans-Differentiation of Neural Stem Cells: A Therapeutic Mechanism Against the Radiation Induced Brain Damage
Source: PLoS One. 2012 Feb 10;7(2):e25936. doi: 10.1371/journal.pone.0025936 (PMC3277599; doi:10.1371/journal.pone.0025936)
Supplement: Table S1 — Oligonucleotide primers and probes used for mRNA expression analysis by real-time PCR. (DOC) [file pone.0025936.s008.doc]

**Table S1.** Oligonucleotide primers and probes used for mRNA expression analysis by real-time PCR

Genes Forward (5' to 3') Reverse (5' to 3')

Ang1 GGGACAGCAGGCAAACAGA TGTCGTTATCAGCATCCTTCGT

CXCL12 CCTCCAAACGCATGCTTCA CCTTCCATTGCAGCATTGGT

FGF2 GTCACGGAAATACTCCAGTTGGT CCGTTTTGGATCCGAGTTTATACT

GAPDH CGTGTTCCTACCCCCAATGT TGTCATCATACTTGGCAGGTTTCT

IGF1 TGCTTCCGGAGCTGTGATCT CGGGCTGCTTTTGTAGGCT

NGF CCCATGGTACAATCCCTTTCA GAACTCCCCCATGTGGAAGA
